# Transcriptomic and Metabolic Analysis Reveals Genes and Pathways Associated with Flesh Pigmentation in Potato (*Solanum tuberosum*) Tubers

**DOI:** 10.3390/cimb46090615

**Published:** 2024-09-17

**Authors:** Man Li, Yuting Xiong, Xueying Yang, Yuliang Gao, Kuihua Li

**Affiliations:** 1 Agricultural College, Yanbian University, Yanji 133002, China; 18626775891@163.com (M.L.); 15971689030@163.com (Y.X.); 17643301229@163.com (X.Y.); 2 Yanbian Agricultural Sciences Academy, Longjing 133400, China; 18943717628@163.com

**Keywords:** potato tuber, anthocyanin, transcriptomics, flavonoid biosynthesis, MYB transcription factors

## Abstract

Anthocyanins, flavonoid pigments, are responsible for the purple and red hues in potato tubers. This study analyzed tubers from four potato cultivars—red RR, purple HJG, yellow QS9, and white JZS8—to elucidate the genetic mechanisms underlying tuber pigmentation. Our transcriptomic analysis identified over 2400 differentially expressed genes between these varieties. Notably, genes within the flavonoid biosynthesis pathway were enriched in HJG and RR compared to the non-pigmented JZS8, correlating with their higher levels of anthocyanin precursors and related substances. Hierarchical clustering revealed inverse expression patterns for the key genes involved in anthocyanin metabolism between pigmented and non-pigmented varieties. Among these, several MYB transcription factors displayed strong co-expression with anthocyanin biosynthetic genes, suggesting a regulatory role. Specifically, the expression of 16 *MYB* genes was validated using qRT-PCR to be markedly higher in pigmented HJG and RR versus JZS8, suggesting that these *MYB* genes might be involved in tuber pigmentation. This study comprehensively analyzed the transcriptome of diverse potato cultivars, highlighting specific genes and metabolic pathways involved in tuber pigmentation. These findings provide potential molecular targets for breeding programs focused on enhancing tuber color.

## 1. Introduction

Anthocyanins are a class of water-soluble pigments responsible for the bright red, purple, and blue hues found in many fruits, vegetables, grains, and flowers [1]. As secondary metabolites, they provide important functions related to plant growth, development, and interaction with the environment [2]. Beyond aesthetics, there is burgeoning interest in anthocyanins for their antioxidant capacity and health-promoting effects in the human diet [3,4]. However, levels tend to be low in commonly consumed foods. Enriching anthocyanin content through crop improvement has thus become an important breeding objective [5]. This review focuses on anthocyanin research in potato (*Solanum tuberosum* L.) tubers, a staple food crop where colored varieties command premium value for their visual appeal and nutritional benefits [6].

Potato tubers exhibit a wide continuum of flesh pigmentation from white to dark purple, attributed to the differential accumulation of anthocyanins [7]. Hundreds of cultivars carry the trait, although levels are often too low for commercial appeal [8]. Understanding the genetic mechanisms controlling tuber coloration will enable the breeding of intensely pigmented varieties to capture their aesthetic and functional advantages. Early crossing experiments indicated simple Mendelian inheritance of flesh color in potato controlled by the P/p locus [9]. White tubers are homozygous pp, whereas pigmented tubers could be PP (intense color) or Pp (intermediate). The P allele was later resolved as a cluster of R2R3 MYB transcription factors on chromosome 10, regulating anthocyanin biosynthesis [10,11]. Additional modifier loci likely assist in finer phenotypic variation [12].

MYBs activate the expression of anthocyanin pathway genes by binding to conserved cis-regulatory motifs in their promoters [13]. The potato anthocyanin genes CHS, F3H, DFR, ANS, and UFGT contain such MYB recognition elements [14]. Co-expression analysis in developing tubers has revealed a close correlation between MYB levels and pigmentation [14], affirming their crucial regulatory role. Transgenic petunia and tomato overexpressing MYBs showed ectopic anthocyanin accumulation [15,16], supporting similar engineering in potato. RNAi-mediated MYB repression reduced foliar pigmentation in potato, although tuber effects were unclear [17]. A combined understanding of the pathway architecture and key controllers is imperative for manipulating anthocyanins in tubers.

The anthocyanin pathway branches off the phenylpropanoid core, diverting flux away from lignin and other branches [18]. Phenylalanine ammonia lyase (PAL) and cinnamate-4-hydroxylase (C4H) provide initial precursors that are converted to dihydroflavonols like dihydrokaempferol by CHS, CHI, and F3H. DFR, ANS, and UFGT then mediate the production of the predominant potato anthocyanins pelargonidin and derivatives [17]. Multiple genes often mediate each step, allowing for fine control over the product spectrum [10,19]. Additional enzymes like AAT acylate anthocyanins enhance their stability [20]. The coordinated up-regulation of this pathway is thus essential for enriching anthocyanins.

While genetics plays a pivotal role, environmental factors like light, temperature, nutrition, and stress also influence tuber anthocyanins [21]. Light signaling via PHYB and HY5 induces MYBs control over photoresponsive pigmentation [22]. Cold temperature boosts anthocyanins in foliage and tubers through CBF/DREB activation [23]. Nitrogen limitation appears to enhance tuber coloration [24]. Wounding and pathogen attacks trigger the production of anthocyanin precursors through PAL [25]. Elucidating these external signals and their integration with genetic regulators is vital to optimizing pigment levels.

The hyperpigmented tuber trait has aroused interest in the nutritional enhancement of potato, a crop that feeds billions globally [26]. Anthocyanin intake lowers risks of cardiovascular diseases, diabetes, neurodegeneration, and certain cancers [27]. Tuber peel is richer in anthocyanins than flesh and shows potent antioxidant capacity [28]. However, levels rapidly decline upon cooking and processing [29]. Engineering stable non-acylated pelargonidins may offer better extractability and bioavailability [30]. Targeted enrichment in tuber plastids can enhance pigment retention [31]. Any tradeoffs with agronomic performance require evaluation under field conditions [32]. Ultimately, the goal is nutritionally augmented potato varieties combining optimized anthocyanin content with high yield, processing quality, and consumer appeal.

In this study, we analyzed tubers from four potato varieties exhibiting differential flesh pigmentation. Integrated metabolite profiling and RNA sequencing revealed the induction of the anthocyanin pathway underlying color enrichment. Several MYB regulators were identified as potential controllers of the pathway based on the expression patterns. These results advance our understanding of the molecular mechanisms regulating anthocyanin accumulation in tubers.

## 2. Materials and Methods

### 2.1. Plant Materials

Germinated seed tubers (10 g) of four potato (*S. tuberosum*) varieties—HJG (heijinggang, purple pulp), RR (red rose, red pulp), QS9 (qingshu No. 9, yellow pulp), and JZS8 (jizhangshu No. 8, white pulp)—were planted in plastic pots (35 cm diameter, 45 cm height) containing a potting mix of peat moss, vermiculite, and perlite (3:1:1 *v*/*v*) in a greenhouse at Yanbian University, Yanji, China. The greenhouse conditions were controlled with a 16 h day/8 h night photoperiod and a 25 °C/18 °C day/night temperature cycle. The pots were watered daily to field capacity and fertilized weekly with a balanced NPK fertilizer (15:15:15). After 50 days of growth, the potato tubers were harvested. Pulp tissue samples (1 cm^3^) were collected from the central region of three randomly selected tubers from each variety. The 12 samples (from different plastic pots) were immediately frozen in liquid nitrogen after being finely chopped, stored at −80 °C, and later used for the next research. This experimental design provided biological replication to account for plant-to-plant variability within each potato variety.

### 2.2. Spectrophotometric Analysis of Bioactive Compounds

Anthocyanins were extracted from four types of potato tubers using a modified [33] method. This involved grinding 1 g samples of the tubers, extracting them twice with a leaching solution at 60 °C, and then centrifuging. The anthocyanin content was measured via visible spectroscopy and calculated using a pH differential method. The total proanthocyanidin content in the pod extracts was measured using the vanillin–hydrochloric acid method after ultrasonic extraction under specific conditions. The process involved centrifugation, the addition of vanillin and hydrochloric acid, and incubation, with the absorbance being measured at 500 nm to calculate the proanthocyanidin content based on a standard curve [34]. The total carotenoid content in the potato tubers was assessed by grinding 5 g samples and extracting with an acetone–petroleum ether mix until colorless, then measuring the absorbance at 451 nm to estimate carotenoids in mg/100 g DW [35]. The total phenolic content in four potato samples was determined using the Plant Total Phenolics Assay Kit (Catalog No: TP-1-G, Keming, Suzhou, China). The absorbance was measured at 760 nm to obtain the total phenolic content of the samples. GA solutions were prepared and used to generate a standard curve. The sample was purified, processed, and analyzed by spectrophotometry against the standard curve to determine polyphenol concentration [36]. Rutin standards were used to generate a calibration curve for spectrophotometric analysis. The sample was then prepared and analyzed against the curve to quantify total flavonoids [36].

### 2.3. RNA Extraction and Sequencing

Total RNA was extracted from four differently colored potato tuber samples using the Trizol reagent for transcriptomic sequencing. The details of the RNA extraction protocol, library preparation protocol, and sequencing technology were derived from Zhao’s study [37]. The construction of the cDNA library, quality control, and sequencing of the RNA-Seq library were carried out by Shanghai Keyi Biotechnology Co., Ltd. We employed the Salmon package (v1.10.3) [38] to map the reads of all samples to the potato reference genome (SolTub_3.0, http://plants.ensembl.org/Solanum_tuberosum/Info/Index, accessed on 3 May 2024). The number of reads mapped to each gene was quantified using the Salmon package. Based on the mapping results from Salmon, the expression levels of genes in all samples were calculated as transcripts per million reads (TPM). To understand the reproducibility among samples, we initially conducted a principal component analysis (PCA) of the transcriptomic data using the PCAtools package (https://bioconductor.org/packages/PCAtools, accessed on 3 May 2024) in R. This included calculating the Pearson correlation coefficient between two samples and presenting the correlation coefficients in the form of a heatmap.

### 2.4. Differential Expression Analysis

Differential analysis of the transcriptomic data was performed using the DESeq2 package (v1.18). To increase the credibility of differentially expressed genes (DEGs), the criteria were set as follows: log2Foldchange > 1.5 and adj.*p*-value < 0.05.

### 2.5. Functional and Pathway Enrichment

To further elucidate the functions and metabolic pathways associated with differentially expressed genes, we performed Gene Ontology (GO, https://geneontology.org/) functional enrichment analysis and Kyoto Encyclopedia of Genes and Genomes (KEGG, https://www.genome.jp/kegg/, accessed on 3 May 2024) pathway analysis on the DEGs. The potato protein sequences were annotated using the Eggnog database, and the annotation results were subjected to GO analysis using the clusterProfiler package (v4.0) in R [39], with the thresholds set at *q*valueCutoff = 0.05 and adj.*p*valueCutoff = 0.05. The enrichment results were visualized using the ggplot2 package in R. The same methodology was applied for the visualization analysis of the KEGG pathways.

### 2.6. Hierarchical Clustering and Co-Expression Analysis

To reveal patterns in the gene expression data across samples, we performed hierarchical clustering analysis using the k-means algorithm as implemented in the ClusterGVis package (https://github.com/junjunlab/ClusterGVis accessed on 3 May 2024). This unsupervised analysis grouped samples together based on the similarity of their overall gene expression profiles. We specifically examined the clustering of expression data for the genes involved in the anthocyanin biosynthesis pathway using heatmaps generated with the Pheatmap package (https://cran.r-project.org/web/packages/pheatmap/index.html accessed on 3 May 2024). This visualization allowed us to assess coordinated expression changes across multiple pathway genes. Additionally, we used the corrplot package (https://github.com/taiyun/corrplot accessed on 3 May 2024) to calculate Pearson correlation coefficients between expression levels of putative transcription factor genes and anthocyanin structural genes across all samples. The results were displayed as a correlational heatmap to identify putative regulatory relationships based on strong positive correlations, which may suggest transcriptional activation.

### 2.7. qRT-PCR Analysis

To validate RNA sequencing outcomes, sixteen members of the *MYB* gene family were selected for verification, and their relative expression levels across different potato varieties were analyzed using qRT-PCR. Primer design was facilitated using Primer 5 software, as detailed in Table S1, with primers supplied by Sangon Bioscience Co., Ltd. (Shanghai, China). Each qPCR reaction contains 10 μL 2 × ChamQ Universal SYBR qPCR Master Mix (Keyi Biosciences Co., Ltd., Shanghai, China), 0.5 μL forward primer, 0.5 μL reverse primer, and 1 μL cDNA. Finally, sterile ddH_2_O is added to 20 μL. The qRT-PCR methodology was adapted from Mo’s research [40].

## 3. Results

### 3.1. Difference Analysis of Physiological Indexes of Potato Tubers with Different Colors

**Figure 1:**
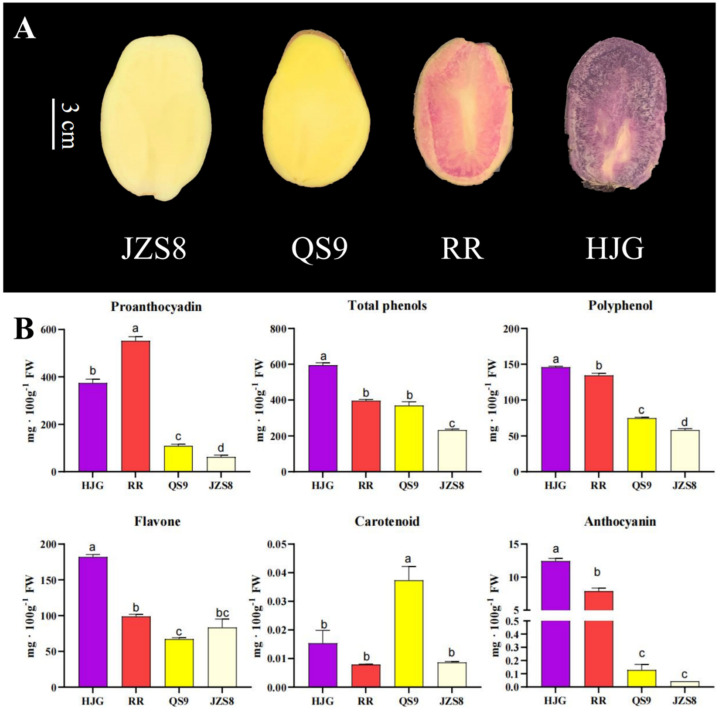
Determination of color and substance content of potato tubers. (**A**) is a physical map of potato tubers of four different varieties. (**B**) is the substance content in tubers of different potato varieties. The contents of proanthocyadin, anthocyanin, total phenols, polyphenol, flavone, carotenoid, and other substances were determined. One-way ANOVA was used to analyze the differential content of the substance. The different letter means *p* < 0.05, error bar = mean ± SD (n = 3).

In this study, the contents of anthocyanin synthesis-related substances in four different varieties of potato materials (RR (red pulp), HJG (purple pulp), QS9 (yellow pulp), JZS8 (white pulp)) were determined (Figure 1A,B). We analyzed the differences in the content of proanthocyanidins, which are precursors of anthocyanins. Among the above four materials, RR and HJG had the highest proanthocyanidin content, and the proanthocyanidin content of RR was higher than that of HJG, but that of HJG was higher than that of RR. The anthocyanin contents of RR and HJG were also significantly higher than those of QS9 and JZS8. Compared with other materials, HJG contains the most polyphenol content, total phenol content, and flavonoid content, followed by RR; QS9 and JZS8 have the same level. The carotenoid content is the highest in QS9, and RR, HJG, and JZS8 are significantly lower than QS9. Through the analysis of physiological indicators and the color of potato tubers, it can be concluded that the red and purple color of potato tubers are directly related to flavonoids and phenolic substances. The formation of yellow tubers was more strongly associated with the accumulation of carotenoids in addition to the phenylpropanoid pathway.

### 3.2. Difference Analysis among Different Samples

**Figure 2:**
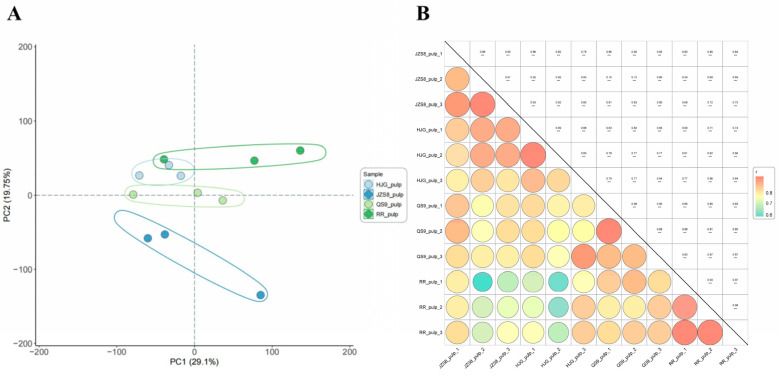
Principal component analysis and correlation cluster analysis between samples. (**A**) is principal component analysis, and (**B**) is correlation cluster analysis, *** means high significant.

Principal component analysis (PCA) reduces the dimensionality of complex transcriptome data containing a large number of gene expression levels and can reflect the regularity between samples to the greatest extent. In this study, PCA was performed on the transcriptome data, and the results showed that there was good separation between the samples in each group (Figure 2A). The PCA plot depicts the sample distribution along the first two principal components, PC1 and PC2, explaining 29.1% and 19.75% of the variance, respectively. We can observe a clear separation of the four potato groups based on their transcriptomic profiles (by PC2); this agrees with the pigmentation phenotypes: HJG and RR being pigmented, while JZS8 and QS9 lack anthocyanins. In addition, cluster analysis results showed a higher correlation among samples within the group (Figure 2B). These results demonstrate the discriminability of transcript data from different materials with good reproducibility within the same material.

### 3.3. Analysis of Differentially Expressed Genes

**Figure 3:**
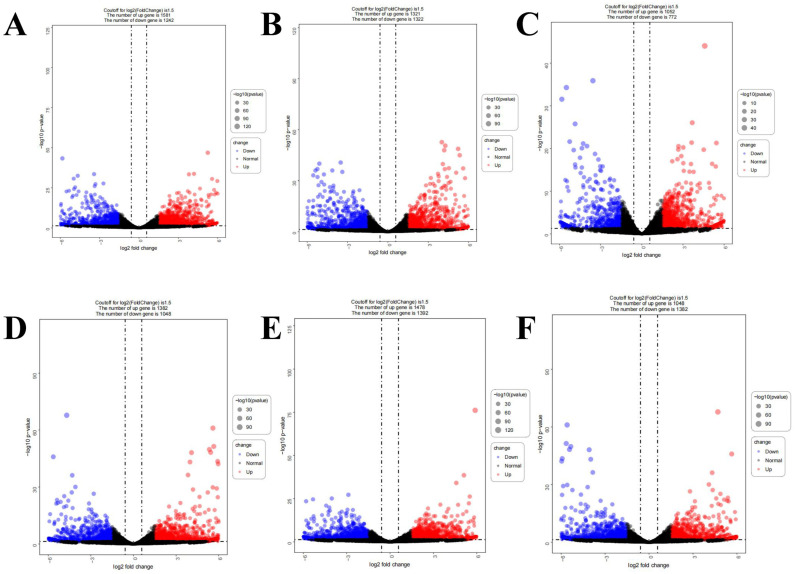
Analysis of differentially expressed genes among tubers of different potato varieties. adj.*p*-value < 0.05 and log2Foldchange > 1.5 is considered to up-regulate expression, marked with red dots; adj.*p*-value < 0.05 and log2Foldchange < −1.5 is considered to down-regulate expression, marked with blue dots. (**A**): HJG_ vs. _JZS8, (**B**): HJG_vs._QS9, (**C**): HJG_ vs. _RR, (**D**): QS9_ vs. _JZS8, (**E**): RR_ vs. _JZS8, (**F**): RR_ vs. _QS9.

In this study, differentially expressed gene analysis (DEG) was performed on two groups of four different potato varieties (Figure 3). A total of 2823 differentially expressed genes were detected between HJG and JZS8, including 1581 up-regulated and 1242 down-regulated. A total of 2643 differentially expressed genes were detected between HJG and QS9, including 1321 up-regulated genes and 1322 down-regulated genes. A total of 2430 differentially expressed genes were detected between QS9 and JZS8, of which 1382 were up-regulated and 1048 were down-regulated. Among the above six groups, the most differentially expressed genes were detected between RR and JZS8, a total of 2870 differentially expressed genes, 1478 up-regulated and 1392 down-regulated. There were 2430 differentially expressed genes between RR and QS9, of which 1048 genes were up-regulated and 1382 genes were down-regulated. It is worth noting that the number of differentially expressed genes detected between the HJG and RR groups was the least, a total of 1824, including 1052 up-regulated genes and 772 down-regulated genes.

### 3.4. Function Analysis

KEGG pathway and GO analyses were performed in order to determine the potential biological functions and metabolic pathways associated with the DEGs. GO can be divided into three major categories, namely, biological process, cellular component, and molecular function (Figure S1). The functional abundance of cortisol synthesis and secretion, steroid hormone biosynthesis, and linoleic acid metabolism of HJG was significantly higher than that of JZS8 (adj.*p* < 0.05). The abundance of plant–pathogen interaction, mismatch repair, homologous recombination, DNA replication, and nucleotide excision repair of RR is higher than that of JZS8 (adj.*p* < 0.05). The GO enrichment analysis results show the functional abundance of response to high light intensity, response to light intensity, tetraterpenoid biosynthetic process, tetraterpenoid metabolic process, carotenoid biosynthetic process, carotenoid metabolic process, Plastoglobules, and chloroplast thylakoid membrane of QS9 lower than that of JZS8 (adj.*p* < 0.05). The abundance of secondary metabolic process in QS9 was significantly higher than that in JZS8 (adj.*p* < 0.05). In RR samples, the abundances of secondary metabolic process, activation of innate immune response, positive regulation of innate immune response, positive regulation of defense response, plant-type hypersensitive response, activation of immune response, and programmed cell death induced by symbiont were lower than those of QS9 (adj.*p* < 0.05). The abundance of response to high light intensity, response to light intensity, tetraterpenoid biosynthetic process, tetraterpenoid metabolic process, carotenoid biosynthetic process carotenoid synthesis, and carotenoid metabolic process carotenoid metabolism of RR was higher than that of QS9 (adj.*p* < 0.05). Crucially, the results of KEGG enrichment analysis showed that HJG and RR had higher enrichment in the flavonoid biosynthesis pathway than JZS8, and the enrichment difference between RR and JZS8 reached a significant level (adj.*p* < 0.05). In addition, there was no significant difference in the enrichment of the flavonoid synthesis pathway between QS9 and JZS8, between RR and QS9 (adj.*p* < 0.05).

### 3.5. Hierarchical Cluster Analysis

**Figure 4:**
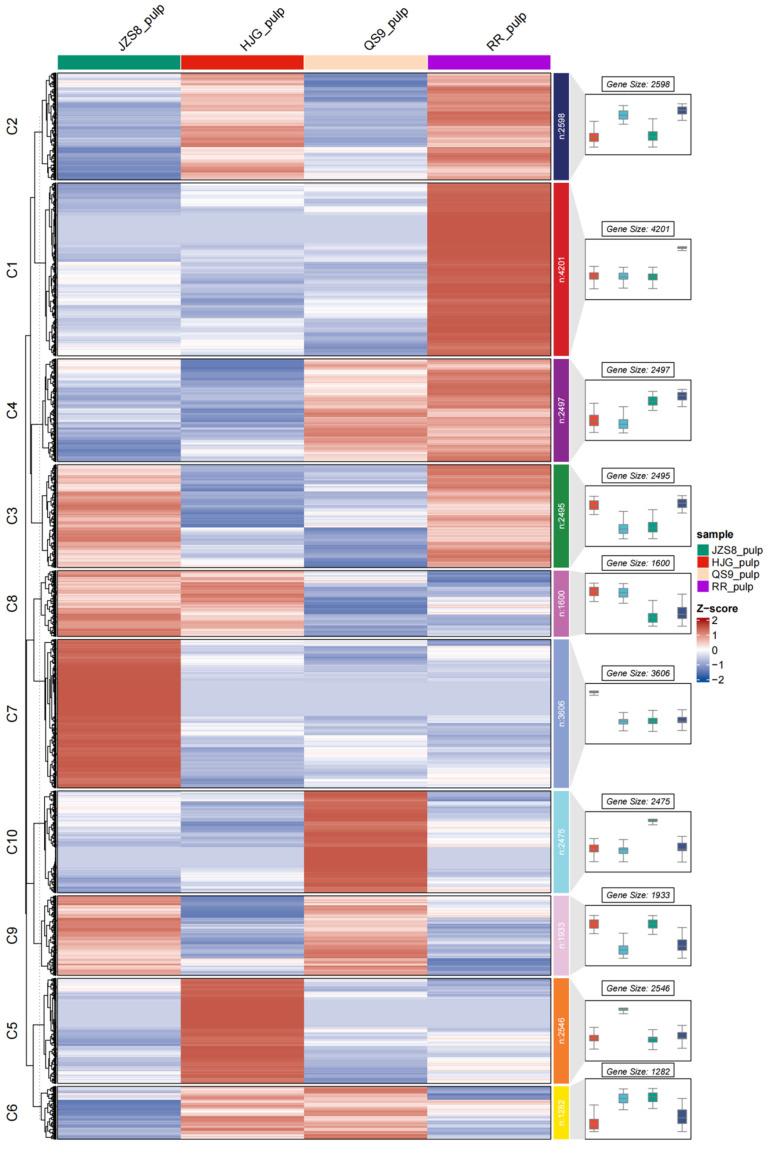
Hierarchical cluster analysis of gene expression in tubers of different potato varieties.

**Figure 5:**
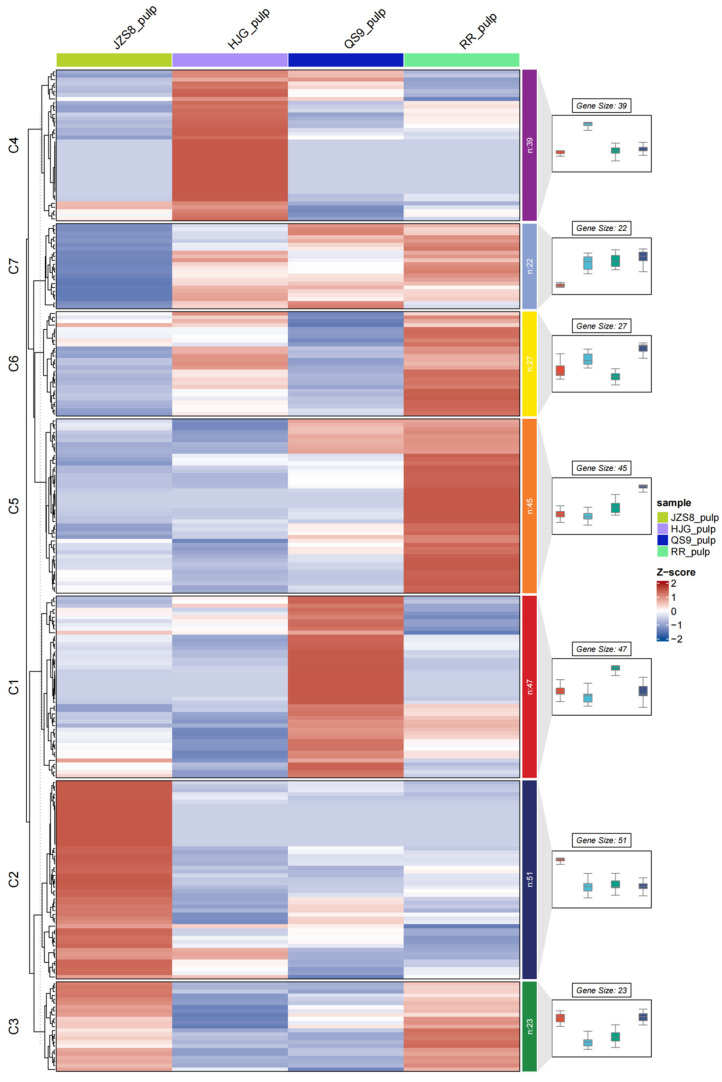
Hierarchical clustering analysis of expression levels of *StMYBs* in different varieties of potato.

Hierarchical clustering analysis can cluster genes with similar expression patterns in the transcriptome into the same gene cluster for analysis and screening of genes with consistent expression in time, tissue, etc., and genes in clusters are more likely to have functional similarities. In this study, a hierarchical clustering analysis was performed according to the expression levels of differentially expressed genes in four different materials, and all genes were divided into 10 clusters (Figure 4). There were 2598 genes and 1933 genes in C2 and C9, respectively, showing consistent expression patterns, and the trends between the two clusters were opposite. In addition, the expression trends of genes in C2 and C9 showed an inverse correlation (positive or negative correlation) with pigment accumulation. Studies have found that the *MYBs* gene family is an important transcription factor family that regulates anthocyanin synthesis in plants. Using the same method, this study conducted a hierarchical clustering analysis on the expression of 255 *StMYBs* genes in four different materials (Figure 5). The results indicated that genes in C2 might be more influential in pigment accumulation in potato tubers.

### 3.6. Analysis of Anthocyanin Synthesis Pathway

**Figure 6:**
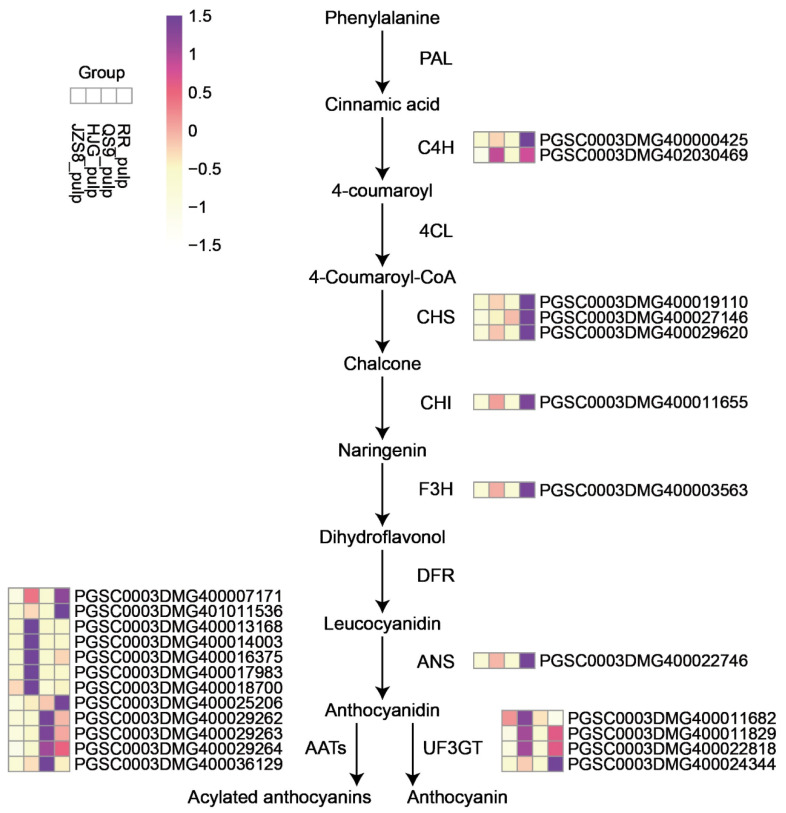
Expression analysis of genes related to anthocyanin biosynthesis metabolic pathway. The expression levels are normalized by row, and the color of the squares gradually darkens as the expression level increases.

**Figure 7:**
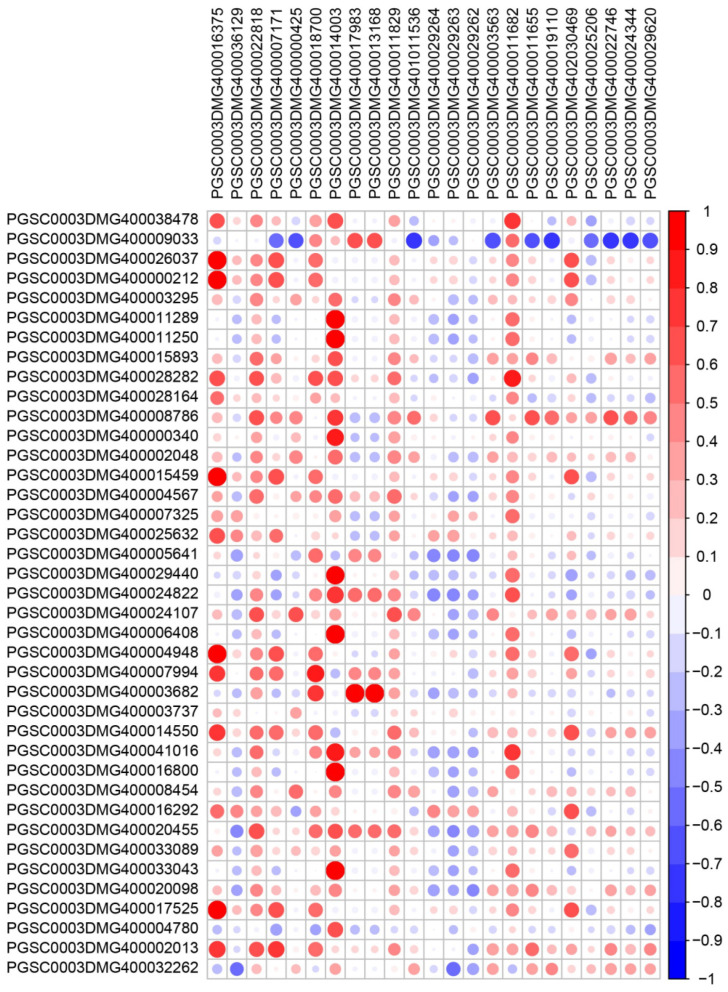
Co-expression analysis of genes related to anthocyanin biosynthesis and *StMYBs*. The level of correlation is proportional to the size and color of the circle, with red representing positive correlation and blue representing negative correlation. The labels on the vertical axis are *StMYBs*, and the labels on the horizontal axis are genes related to anthocyanin synthesis.

Based on the genome annotation file (gff3 file of SolTub_3.0), we found some genes associated with anthocyanin biosynthesis pathway. Phenylalanine is deaminated by phenylalanine ammonia-lyase (PAL) and hydroxylated by cinnamate-4-hydroxylase (C4H) to trans-4-coumaric acid. This study found that the C4H synthesis genes *PGSC0003DMG400000425* and *PGSC0003DMG402030469* were highly expressed in HJG and RR, which promoted the initial step of anthocyanin synthesis (Figure 6). In the subsequent anthocyanin biosynthesis pathway, CHS synthesis genes (*PGSC0003DMG400019110*, *PGSC0003DMG400027146* and *PGSC0003DMG400029620*), CHI synthesis genes (*PGSC0003DMG400011655*), and F3H synthesis genes (*PGSC0003DMG400003563*) were found in HJG and Expression levels were higher in RR, which promoted the accumulation of leucocyanin (Figure 7). The high expression of an ANS synthesis gene (*PGSC0003DMG400022746*) promoted the synthesis of colored anthocyanins in HJG and RR. Subsequently, anthocyanins formed anthocyanins and acylated anthocyanins under the action of UF3GT and AATs, which improved the color stability. In HJG and RR, UF3GT synthetic genes (*PGSC0003DMG400011682*, PGSC0003DMG400011829, PGSC0003DMG400022818, PGSC0003DMG400024344) and AATs synthetic genes (*PGSC0003DMG400007171*, *PGSC0003DMG401011536*, *PGSC0003DMG400013168*, *PGSC0003DMG400014003*, *PGSC0003DMG400016375*, *PGSC0003DMG400017983*, *PGSC0003DMG400018700*, *PGSC0003DMG400025206*, *PGSC0003DMG400029262*, *PGSC0003DMG400029263*, *PGSC0003DMG400029264*, *PGSC0003DMG400036129*) have higher expression levels, indicating that these two varieties of potatoes are more likely to promote stable pigment synthesis (Figure 7).

In this study, the co-expression analyses of *MYB* genes and genes related to anthocyanin synthesis were carried out (Figure 7). *PGSC0003DMG400016375*, *PGSC0003DMG400022818*, *PGSC0003DMG400007171*, *PGSC0003DMG400018700*, *PGSC0003DMG400014003* and *MYBs* genes (*PGSC0003DMG400026037*, *PGSC0003DMG400000212*, *PGSC0003DMG400028282*, *PGSC0003DMG400008786*, *PGSC0003DMG400015459*, *PGSC0003DMG400004948*, *PGSC0003DMG400007994*, *PGSC0003DMG400014550*, *PGSC0003DMG400017525* and *PGSC0003DMG400002013*) have a significant positive correlation. *PGSC0003DMG400014003*, *PGSC0003DMG400011829*, *PGSC0003DMG400011682* are strongly positively correlated with most *MYBs*. This suggests that the formation of potato tuber color might be determined by MYBs-regulated anthocyanin biosynthesis.

### 3.7. qRT-PCR Analysis of MYB Genes

**Figure 8:**
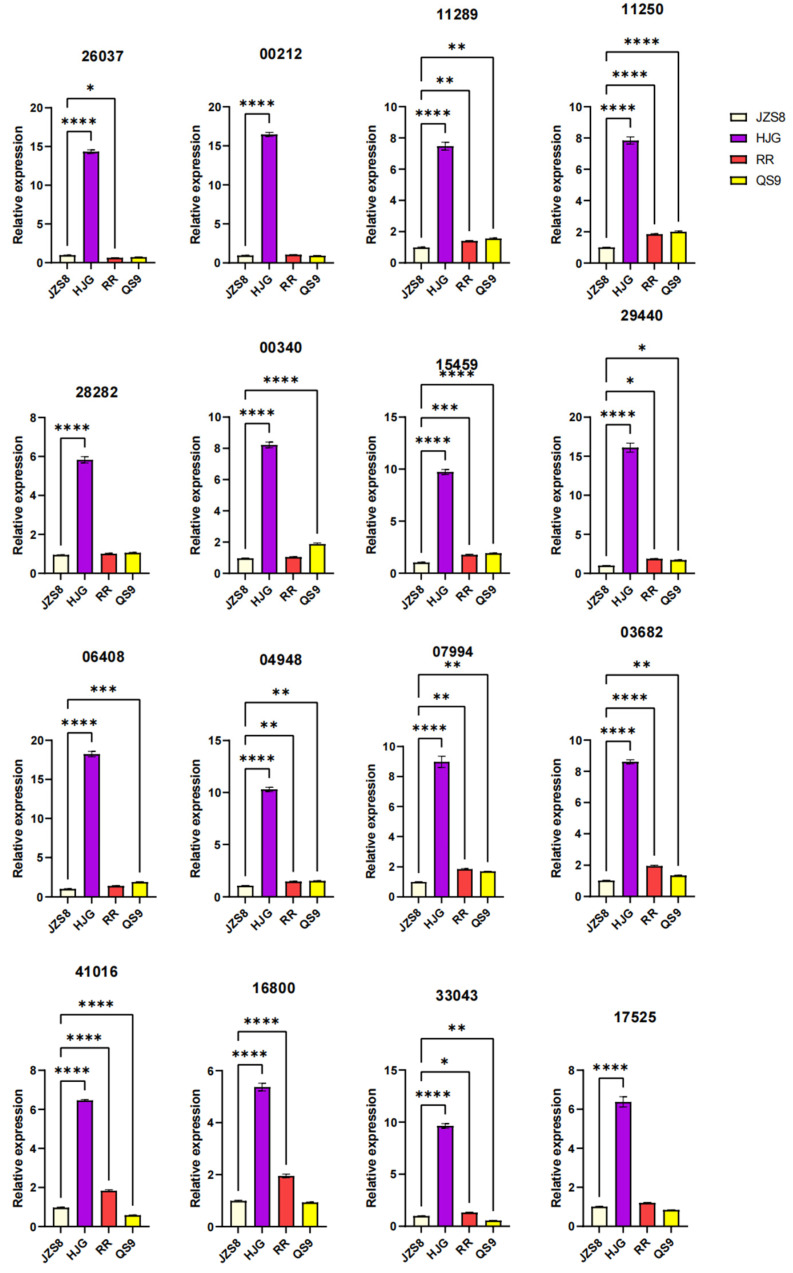
qRT-PCR analysis of MYB genes in four potato varieties. For simplicity, the character “PGSC0003DMG4000” in the gene ID has been omitted from the figure. One-way ANOVA was used to analyze the differential expression of genes. The number of * denotes different *p*-values, * means *p* < 0.05, ** means *p* < 0.01, *** means *p* < 0.001 and **** means *p* < 0.0001. Error bar = mean ± SD (n = 3).

In our study, we focused on 16 *MYB* genes selected based on their high correlation with anthocyanin biosynthesis-related genes. This selection was informed by co-expression analysis, where these *MYB* genes exhibited correlation coefficients greater than 0.9, with genes directly involved in anthocyanin synthesis. This strong correlation suggests a potential regulatory role in the anthocyanin biosynthetic pathway. The expression levels of these 16 *MYB* genes (*PGSC0003DMG400026037*, *PGSC0003DMG400028282*, *PGSC0003DMG400006408*, *PGSC0003DMG400041016*, *PGSC0003DMG400000212*, *PGSC0003DMG400000340*, *PGSC0003DMG400004948*, *PGSC0003DMG400016800*, *PGSC0003DMG400011289*, *PGSC0003DMG400015459*, *PGSC0003DMG400007994*, *PGSC0003DMG400033043*, *PGSC0003DMG400011250*, *PGSC0003DMG400029440*, *PGSC0003DMG400003682*, and *PGSC0003DMG400017525*) were quantitatively analyzed across different potato cultivars (Figure 8). Notably, genes such as *PGSC0003DMG400026037*, *PGSC0003DMG400028282*, *PGSC0003DMG400006408*, and *PGSC0003DMG400041016* showed substantially higher expression levels in the purple HJG and red RR cultivars compared to JZS8. This elevated expression in colored potato varieties, particularly in those rich in anthocyanins (HJG and RR), reinforces the potential role of these *MYB* genes in modulating anthocyanin production and, consequently, the flesh color of potatoes. These results highlight the importance of MYB transcription factors in the anthocyanin biosynthesis pathway and their potential as key molecular targets for the genetic manipulation and breeding of potato cultivars with specific color attributes.

## 4. Discussion

This study provided extensive metabolite patterns and transcriptomic insights into potato tubers exhibiting differential flesh pigmentation. Anthocyanin profiling revealed stepwise enrichment in red and purple varieties, underpinned by stimulation of the phenylpropanoid pathway based on gene expression patterns. Co-expression analysis hinted at the coordinated regulation of structural genes by MYB transcription factors. These results significantly advance understanding of anthocyanin accumulation in tubers and identify putative regulators for boosting this nutritionally important trait.

Anthocyanins impart bright red/purple hues in many fruits and vegetables, influencing quality attributes and consumer appeal [41]. In potato tubers, flesh pigmentation shows extensive natural variation attributed to allelic diversity and transcriptional control of the flavonoid pathway [14,42]. White and yellow varieties lack anthocyanins, directing flux towards competing branches like carotenoids [43]. This study surveyed tubers with four flesh colors, revealing up to 70-fold higher anthocyanins in red/purple types (Figure 1). Their high levels correlated with the stepwise elevation of precursor compounds like flavonoid intermediates, highlighting the co-induction of the underlying phenylpropanoid pathway. Non-pigmented tubers had reduced phenolics, resulting in pale hues. The metabolite patterns were consistent with known anthocyanin biosynthesis, as discussed below through integrated transcriptomic analysis.

The anthocyanin pathway branches off the phenylpropanoid core at naringenin and involves sequential enzymes like CHS, CHI, F3H, DFR, and ANS [44,45]. Most steps were up-regulated in pigmented tubers based on gene expression levels, constituting a coordinated flavonoid module (Figure 3, Figure 4 and Figure 6). C4H contributes key initial precursors like 4-coumaric acid [46] and showed induction as expected (Figure 6). The expressions of some members of AATs were not consistent with the color trend of potato tubers, which means that there may be differences in the substrate specificity of potatoes [20]. Up-regulation extended to UF3GT and AATs, which enhance color intensity and stability through decorating modifications to anthocyanidins [47,48]. Their induction enables maximal pigment production and retention. Representatives of the coordinated module can serve as metabolic markers for breeding intensely colored varieties.

In addition to structural genes, coordinated regulation is essential for stimulating anthocyanin accumulation. Anthocyanin-related MYB transcription factors activate promoters of pathway genes through conserved R2R3 motifs [45]. Potato genes possess such cis-elements, pointing to MYB-mediated control [49]. This study identified a subset of MYBs showing expression patterns correlated with pigmentation besides positive co-expression with anthocyanin genes (Figure 7). These likely represent activators of the potato tuber flavonoid pathway, reminiscent of characterized MYBs in grape hyacinth [50] and Norway spruce [51]. Their overexpression or activation using genome editing may stimulate anthocyanin production, as demonstrated for kiwifruit [45] and tomato [52]. The MYB-bHLH-WD40 complex model provides a framework for the transcriptional control of anthocyanin genes [13]. bHLH factors form a bridge between MYBs and WD40 proteins to synergistically activate the pathway. Additional MYBs beyond the identified candidates may participate to confer specificity. HY5 transcription factors also assist in light-mediated anthocyanin regulation [53,54]. Future efforts are required to uncover interacting regulatory components influencing tuber pigmentation. Characterizing environmental and hormonal signals and modulating their activity will help establish a dynamic model of anthocyanin control. Anthocyanin enrichment is a breeding target owing to associated health benefits [55]. However, levels in existing pigmented potato cultivars are often too low for commercial viability [56]. This study elucidated candidate MYBs that may stimulate anthocyanin production upon transgenic overexpression or genome editing activation, providing molecular tools for crop improvement. Targeted modification avoids potential drawbacks of whole-pathway engineering like resource tradeoffs. Tuber-specific promoters can focus enhancement on flesh while preventing pigmentation of foliage and other organs. However, stability during processing and storage requires appraisal prior to commercial development [57]; for instance, the development of molecular markers associated with MYB genes plays a crucial role in breeding potatoes with elevated anthocyanin content [58]. Finally, and of considerable importance, not all *MYB* genes in the potato anthocyanin biosynthesis pathway necessarily act as positive regulators. For instance, in *Cynara cardunculus*, *MYB12* has been found to negatively regulate anthocyanin synthesis [59]. Moreover, anthocyanin biosynthesis is also subject to regulation by miRNAs [60]. These factors warrant careful consideration in our efforts to breed potatoes with enhanced anthocyanin content.

## 5. Conclusions

In conclusion, this study performed extensive profiling of potato tubers exhibiting differential flesh pigmentation owing to anthocyanin enrichment. Metabolite patterns were consistent with pathway induction revealed through integrated transcriptomics. Key structural genes showed coordinated up-regulation with candidate MYB regulators, unraveling putative control mechanisms. These novel insights advance fundamental understanding of anthocyanin accumulation in tubers and provide molecular resources to enhance this nutritionally important trait. Further efforts to characterize the identified MYBs along with interacting partners and signals will help establish their applicability in stimulating flavonoid production. Modern breeding approaches can translate these leads into improved varieties with enhanced aesthetic and nutritional qualities.

## Data Availability

The raw data have been uploaded to NCBI with the accession number: PRJNA1151859.

## Supplementary Materials

The following supporting information can be downloaded at https://www.mdpi.com/article/10.3390/cimb46090615/s1, Figure S1: Functional enrichment analysis of different varieties of potato genestitle; Table S1: Primers for this study.
