# Supplementary material for: Transcriptomic and Metabolic Analysis Reveals Genes and Pathways Associated with Flesh Pigmentation in Potato (Solanum tuberosum) Tubers
Source: Curr Issues Mol Biol. 2024 Sep 17;46(9):10335–50. doi: 10.3390/cimb46090615 (PMC11430057; doi:10.3390/cimb46090615)
Supplement: Supplementary file 1 [file cimb-46-00615-s001.zip › cimb-3153832 Figure S1.pdf]

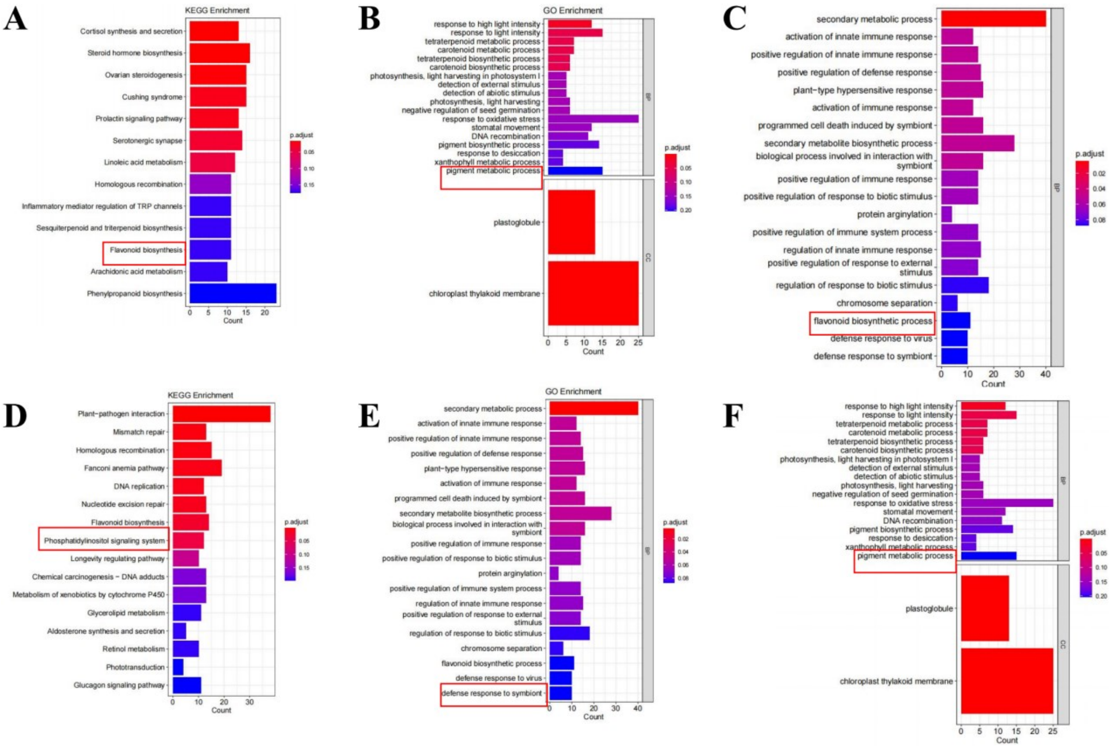

Figure S1. Functional enrichment analysis of different varieties of potato genes. A: HJG vs JZS8 KEGG Down enrichment; B: QS9 vs JZS8 GO Down enrichment; C: QS9 vs JZS8 GO Up enrichment; D: RR vs JZS8 KEGG Down enrichment; E: RR vs QS9 GO Down enrichment; F: RR vs QS9 GO Up enrichment.
